# Age-related changes in the water-soluble lens protein composition of Wistar and accelerated-senescence OXYS rats

**Published:** 2011-06-01

**Authors:** Lyudmila V. Kopylova, Ivan V. Cherepanov, Olga A. Snytnikova, Yuliya V. Rumyantseva, Nataliya G. Kolosova, Yuri P. Tsentalovich, Renad Z. Sagdeev

**Affiliations:** 1 International Tomography Center SB RAS, Institutskaya 3a, Novosibirsk, Russia; 2 Novosibirsk State University, Pirogova 2, Novosibirsk, Russia; 3 Institute of Cytology and Genetics SB RAS, Acad. Lavrentjev 10, Novosibirsk, Russia

## Abstract

**Purpose:**

To determine the age-related and the cataract-specific changes in the crystallin composition in lenses of accelerated-senescence OXYS (cataract model) and Wistar (control) rats.

**Methods:**

The water soluble (WS) and insoluble (WIS) fractions of the lens proteins were separated; the identity and relative abundance of each crystallin in WS fraction were determined with the use of two-dimensional electrophoresis (2-DE) and Matrix-Assisted Laser Desorption Ionization – Time Of Flight (MALDI-TOF) mass spectrometry. All statistical calculations were performed using the software package Statistica 6.0 by factor dispersion analysis (ANOVA/MANOVA) and Newman-Keuls post-hoc test for comparison of group mean values.

**Results:**

The WIS protein content increased significantly in the aged animal lenses; the WIS/WS ratio increases in approximately 8 times to the age of 62 weeks. The interstrain difference was insignificant in this experiment. 2-DE maps of the young rat lenses (3 weeks) showed single spots for each lens protein while in older lenses (12 and 62 weeks) each crystallin was presented by several spots. The abundance of γA-γF-crystallins in WS fraction significantly decreases with age. A significant increase in the percentage abundance was also found for α-crystallins and βB2-crystallin from 3 to 12 weeks. The major differences between Wistar and OXYS lenses are the faster decay of the content of γA-γF-crystallins in OXYS lenses, and the significant decrease of unmodified αA-crystallin abundance in old OXYS lenses.

**Conclusions:**

The presented results demonstrate that the increase of the water-insoluble (WIS) protein fraction is rather age-specific than cataract-specific phenomenon. The major age-related changes in WS protein composition are the fast insolubilization of γ-crystallins, and the increase of αB- and βB2-crystallin abundance. The main interstrain differences, which could be attributed to the cataract-specific processes, are the faster decay of the content of γ-crystallins and the significant decrease of unmodified αA-crystallin abundance in the OXYS lenses.

## Introduction

In spite of the progress made in surgical techniques in many countries during the last ten years, cataracts remain the leading cause of visual impairment globally, particularly in Asia [1]. The conditions of visual diseases are strongly age-related with earlier signs occurring in the middle age, which become more severe and more prevalent with increasing age, especially, under risk factors such as diabetes, smoking, and exposure to sunlight. The “free radical theory” of aging postulates that the major factor leading to senile cataract is the oxidation of the lens tissues by reactive oxygen species (ROS) [2].

Lens is a unique organ because of its composition and properties. Lens proteins constitute more than 30% of the lens total mass [3-5]. The major constituents of the lens are crystallins – the structural proteins that amount to about 90% of the total proteins in the lens [6,7]. There are three types of crystallins in the mammalian lens – α-, β-, and γ-crystallins, with a high level of homology within each group [8]. The transparency of the eye lens strongly depends on the crystallin solubility and structure [9-11]. All crystallins are initially water-soluble (WS). During aging, crystallins may undergo post-translational modifications (PTMs) which accumulate throughout life, since the turnover of crystallins in the lens is negligible [12]. Modifications of the lens crystallins disrupt its normal structure in the lens: proteins undergo oxidation, coloration, aggregation, and lose the solubility. Crystallins start forming large insoluble aggregates that may exceed the dimension of the wavelength of light and produce fluctuations in the refraction index causing light scattering [3,5,13,14]. These processes eventually result in the lens opacification and cause pathological alterations leading to the cataract formation [15]. The well known PTMs are the protein truncation, deamidation, glycation, disulfide formation, cross-linking, and phosphorylation [6,7,10,11,16,17]. The most significant influence on the protein insolubility is probably caused by the PTMs of α-crystallins: in the tissues α-crystallins appear to have chaperone activity which may decrease due to modifications [8,18-21]. Other crystallins undergo similar changes, and all these processes lead to the increasing percent of water-insoluble (WIS) fraction during aging [13,22,23]. In several studies, it was shown that the listed modifications are common for primates and rodents such as mice [24-26] and rats [27-29]. At present, it is not completely clear which modifications are cataract-specific, and which are just a part of the normal maturation and aging processes (such as site-specific proteolysis of β-crystallins in young lenses for tighter packing of crystallins during maturation [17,27]). It is necessary to have both normal and cataractous lenses of different ages to compare the obtained results and to be able to detect disease-specific modifications. Obviously, normal human lenses are difficult to obtain, especially from the young donors. Thus, different rodent models have been developed to study the aging processes [30-34]. None of these models exactly matches to what occurs in the human lens, but the mechanisms of cataract formation are supposed to be similar.

In this study, we used the strain of senescence-accelerated OXYS rats, which appears to be a good model of senile cataract [35-37]. This strain was developed at the Institute of Cytology and Genetics SB RAS, Novosibirsk, Russia, from Wistar stock by selection for their susceptibility to the cataractogenic effect of galactose [38]. It was shown that the first signs of cataract in OXYS rats appear at the age of 1.5 months, to the age of 3 months 90% of animals are affected by the lens opacification, and at the age of 4–6 months the morbidity reaches 100% [36,39]. The mature cataract was detected in 90% of eyes of two-year-old OXYS rats, whereas the same aged Wistar rats showed only the initial signs of cataract [39].

The purpose of the present study was to analyze the water-soluble protein content of OXYS and Wistar rat lenses and to determine its age-dependent alterations. In this work, we obtained 2-DE protein maps for lenses of increasing age and of two rat strains to serve as reference for further investigations. The protein identities were confirmed by mass-spectrometry (MALDI) and each protein relative content was quantified. The obtained data will facilitate the future experiments on detecting the cataract-specific modifications in rat lenses.

## Methods

### Materials and reagents

Phosphate buffer tablets (Biolot, Saint-Petersburg, Russia), urea (Bio-Rad Laboratories, Hercules, CA), acrylamide (4K; Medigen Laboratories, Novosibirsk, Russia), acrylamide for IEF (Amersham Biosciences, Uppsala, Sweden), bis-acrylamide (Amresco, Solon, OH), ampholites (Bio-Lyte 3/10; Bio-Lyte 5/8; Bio-Rad), CHAPS (Bio-Rad), ammonium persulphate (PSA; Helicon, Moscow, Russia), TEMED (Helicon), NaOH (Reachim, Moscow, Russia), orthophosphoric acid (Reachim), glycerol (Panreac, Barcelona, Spain), Tris-HCl (Bio-Rad), glycine (Bio-Rad), SDS (Bio-Rad), DTT (Helicon), bromophenol blue (Helicon), agarose (Bio-Rad), Coomassie brilliant blue R-250 (Sigma, Steinheim, Germany), acetic acid (Chimreactiv, Moscow, Russia), ammonium bicarbonate (AMB; Fluka, Steinheim, Germany), ACN (Cryochrom, Saint-Petersburg, Russia), sequencing grade modified trypsin (Promega, Madison, WI), TFA (Sigma), 2.5-DHB (Bruker Daltonics, Bremen, Germany) were used as received. H_2_O was deionized.

### Rat lens preparation

All animal procedures adhered to the Association for Research in Vision and Ophthalmology (ARVO) statement for the Use of Animals in Ophthalmic and Vision Research and in compliance with the European Communities Council Directive No. 86/609/EES. Lenses were obtained from Wistar and senescent-accelerated OXYS rat strains maintained in the Breeding Laboratory of the Institute of Cytology and Genetics SB RAS, Novosibirsk, Russia. Animals were put to sleep with use of diethyl ester and decapitated. The lenses were removed, frozen in liquid nitrogen, and stored at −70 °C until analysis. The content of the water-soluble and water-insoluble lens proteins was determined using lenses from Wistar and OXYS rats at the age of 3, 12, 54, and 62 weeks. Lenses were homogenized in 600 μl of 0.02 M phosphate buffer solution, pH 7.3, containing protease inhibitor (Protease Inhibitor Cocktail; Sigma). The WS and WIS fractions were separated by centrifugation at 12,000× g for 50 min at 4 °C. After separation, the supernatant and the pellet were dried in a vacuum evaporator (Christ AVC 2–25 CD Plus; 3000 g; 37 °C;  Martin Christ Gefriertrochnungsanlagen GmbH, Osterode am Harz, Germany), and then weighted. Clean buffer solution was also dried and weighted, and the mass of the buffer salts was subtracted from the masses of WS and WIS protein fractions.

The WS proteins for electrophoresis were extracted from 36 lenses from Wistar and OXYS rats at the age of 3, 12, and 62 weeks. The number of animals in each group varied from 3 to 6. The proteins were isolated from lenses by homogenization on ice in 0.02 M phosphate buffer solution, pH 7.3, containing protease inhibitor (Protease Inhibitor Cocktail; Sigma). The volume of the buffer solution for 12- and 62-week-old rats was 600 μl per lens (only one lens from each animal was used). Since 3-week-old lenses are much smaller than the mature ones, the homogenization was performed using both lenses from each animal of this age, and the buffer solution volume was 300 μl per lens pair. The WS and WIS fractions were separated by centrifugation at 12,000× g for 50 min at 4 °C. The protein content in supernatants was determined using Bradford reagent [40] (Fermentas Inc., Glen Burnie, MD) and BSA standard (Bio-Rad) following the manufacturer’s protocol.

RNA isolation, reverse transcription, preparation of “standard” cDNA and real time PCR were described in details earlier [39]. Briefly, total cell RNA was isolated from the rat lens by the phenol–chloroform method. Expression of alpha-crystallin A chain (*Cryaa*) and alpha-crystallin B chain (*Cryab*) genes was determined by real time PCR, the housekeeping gene 60S ribosomal protein L30 (*Rpl30*) was used as a reference gene.

### Two-dimensional gel electrophoresis

Isoelectric focusing (IEF) was performed using “tube gel” system. The gel for the first dimension contained 1.75 g urea, 1,190 μl H_2_O, 455 μl of stock solution containing 30% acrylamide for IEF and 0.8% bis-acrylamide, 62 μl ampholites 3/10, 123 μl ampholites 5/8, 210 μl 30% CHAPS, 7 μl 10% PSA (freshly prepared), and 3.5 μl TEMED. The final percentage of acrylamide in tube gels was approximately 4%. PSA and TEMED were added to the solution directly prior casting the gel. The gels were cast in glass tubes (length 20 cm, internal diameter 1.5 mm). The length of gels in tubes was 18 cm. After the gel polymerization, 150 μg (in 10 μl) of WS protein mixture (prepared as written above) from each sample was loaded onto the top of the gels followed by 0.05 M NaOH (~10 μl) to fill the tube up. The bottom of the tube was covered with a drop of 0.13% orthophosphoric acid to dislodge any bubbles from inside the gel tubes. The tube stand with the tubes was fixed on a cooling core and placed into the lower tank of a Protean II xi 2-D Cell (Bio-Rad) filled with 0.13% orthophosphoric acid. The upper tank was filled with 400 ml of 0.05 M NaOH. Running conditions for IEF were: 100 V to 600 V over 1 h, 700 V over 10 h, 900 V over 1 h, room temperature.

After the IEF stage, the gels were extruded from the tubes into the tray with buffer solution containing 6 M urea, 30% glycerol, 12.5 mM Tris-HCl (pH 6.8), 2% SDS, a trace of bromophenol blue; 0.2% DTT was added immediately before use. The gels were left in the solution for 5–10 min, and then placed over the 12% SDS–PAGE gel for the second dimension. The gels were covered with 0.9% agarose containing a trace of bromophenol blue tracking dye. Slabs for the second dimension were prepared in advance. SDS–PAGE gels (12%; 20×20 cm, thickness 1.5 mm) contained 120 ml of stock solution (30% acrylamide and 0.8% bis-acrylamide), 1.5 M Tris-HCl with 0.4% SDS (76 ml), 10% PSA (682 μl), TEMED (123 μl) and H_2_O (103 ml). The prepared slabs were fixed on a cooling core and placed into the lower tank of a Protean II Multi-Cell (Bio-Rad) filled with electrophoresis buffer (25 mM Tris (pH 8.3), 192 mM glycine, 0.1% SDS). Running conditions for the second dimension were: 20 mA per gel (for 20 min), 40 mA per gel (for 2 h), 35 mA per gel (for 3 h). After electrophoresis, the gels were stained with 0.025% Coomassie blue R-250 in 10% acetic acid.

### Protein quantification and identification

Gel images were obtained using a VersaDoc Imaging System (4000 MP; Bio-Rad), and calculation of the protein percentage abundances was performed with a PDQuest Advanced 2D-analysis Software 8.0.1 (Bio-Rad). Proteins from gels were identified by MS analysis. Coomassie stained spots from gels (for each strain and each age of animals) were manually excised (including negative control, no protein) and washed twice in 0.2 M AMB buffer in 50% ACN at 37 °C to remove the stain. Each gel piece was dried with 100% ACN and digested with sequencing grade modified trypsin (12.5 ng/μl) plus 40 mM AMB for 16 h at 37 °C. Peptides were desalted using C18 ZipTips (Millipore Corporation, Billerica, MA), mixed (in a ratio of 0.5 μl of the sample to 0.5 μl of the matrix) with 2.5-DHB dissolved in 70% ACN/0.1% TFA and spotted to a standard MTP ground steel plate (Bruker Daltonics, Bremen, Germany). The further analysis was performed using a MALDI-TOF/TOF spectrometer Ultraflex III (Bruker Daltonics). The mass spectra of protein tryptic digests were recorded in reflective positive ion mode in the 500–4200 m/z range. Spectra were then analyzed using a FlexAnalysis software 3.0 (Build 96; Bruker Daltonics), and peptide masses were entered into the local MASCOT server 2.2.04 for the identification of peptides. The MALDI-TOF identities of proteins were established by using the SwissProt database (updated 2010–08–10; mass accuracy – 70 ppm, 1 missed cleavage).

### Statistical analysis

All statistical calculations were made using the software package Statistica 6.0 (Statsoft Russia, Moscow, Russia) using factor dispersion analysis (ANOVA/MANOVA) and Newman-Keuls post-hoc test for comparison of group mean values. Genotype and age of animals were considered as independent factors. In all cases, results were considered as statistically significant at p<0.05.

## Results

### Age-related changes in WIS/WS protein ratio

The measurements of the WS and WIS protein content in the lens of OXYS and Wistar rats of different age were performed for lenses of three animals for every age and every strain. The analysis was performed for every lens separately, the averaged results are presented in Table 1. For both strains, a significant increase of the WIS protein content is observed: the WIS/WS ratio increases from approximately 0.1 (3 weeks), to 0.4 (12 weeks), to 0.8 (62 weeks). The interstrain difference for all ages is insignificant: in fact, for all ages the WIS/WS ratio for Wistar rats is slightly higher than that for senescence-accelerated OXYS rats, but this difference is below the experimental error.

**Table 1:** Content of WS and WIS proteins in lenses of Wistar and OXYS rats.

**Age**	**Strain**	**Lens weight, mg**	**WS proteins mass, mg**	**WIS proteins mass, mg**	**Ratio WIS/WS**
3 weeks	Wistar	16.0±0.7	5.8±0.2	0.65±0.13	0.11
	OXYS	17.8±0.9	6.3±0.2	0.64±0.15	0.10
12 weeks	Wistar	37.6±1.9	12.9±1.6	4.9±0.2	0.38
	OXYS	41.7±2.1	13.9±0.6	4.3±0.4	0.31
54 weeks	Wistar	69.3±4.3	21.4±1.6	12.7±1.6	0.60
	OXYS	68.5±2.4	21.4±1.0	11.4±0.5	0.53
62 weeks	Wistar	79.4±4.4	18.6±1.1	15.9±1.5	0.85
	OXYS	64.3±5.2	16.3±1.7	12.9±0.9	0.79

### 2-DE maps of water-soluble proteins

**Figure 1:**
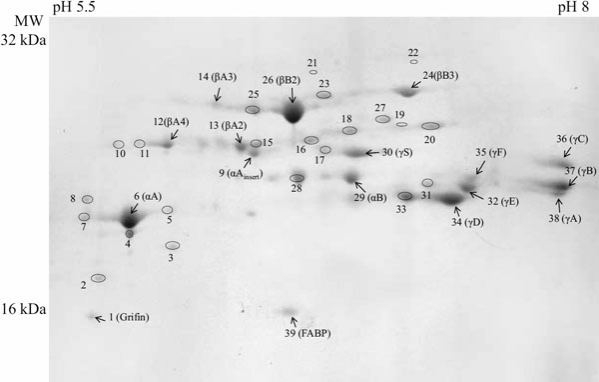
2-DE gel of the lens proteins of 12-week-old Wistar rat stained with Coomassie brilliant blue R-250. Arrows indicate the unmodified proteins, and their assignment is shown, the modified crystallins are given the numbers only: spots 2–5,7,8 correspond to αA-crystallin, spots 10,11 – to βA4-crystallin, spots 15–20 – to βA3-crystallin, spot 22 – to βB1-crystallin, spot 23 – to βB3-crystallin, spots 26–27 – to βB2-crystallin, spot 28 – to αB-crystallin, spot 31 – to γE-crystallin and spot 33 – to γD-crystallin. The assignment of all indicated spots and the sequence coverage are also presented in Table 2.

To determine the relative abundance, the identities of crystallin subunits, and the age-related differences between OXYS and Wistar rat strains, 2-DE was performed, the spots were visualized and identified by in-gel digestion with the subsequent MS analysis. Figure 1 shows a 2-DE map of the water-soluble protein fraction from the lens of a 12-week-old Wistar rat, the assignments of the major proteins are indicated. The identities of proteins were determined with 60%–80% of sequence coverage by MALDI-MS analysis of tryptic peptides. The results obtained for the WS fraction of 12-week-old Wistar lens are given in Table 2, the same procedure was applied to proteins of all ages and both strains. In 2-DE maps of 3-week-old lenses, most of the crystallins are represented by a single spot, while in older lenses the number of spots significantly increases. Thus, the spots present in the maps of young lenses are attributed to the unmodified proteins, while the new spots observed in the maps of older lenses correspond to the crystallins, which underwent post-translational modifications. In Figure 1, arrows indicate the unmodified proteins, and their assignment is shown, whereas the modified crystallins are given the numbers only: spots 2–5, 7, and 8 correspond to modified αA-crystallins, spots 10–11 – to modified βA4-crystallins, spots 15–20 – to modified βA3-crystallins, spot 22 – to modified βB1-crystallin, spot 23 – to modified βB3-crystallin, spots 26–27 – to modified βB2-crystallins, spot 28 – to modified αB-crystallin, spot 31 – to modified γE-crystallin and spot 33 – to modified γD-crystallin. The obtained 2-DE maps are in a good agreement with the crystallin maps of rats and mice [25,29] published earlier. In addition, we were able to obtain a good resolution for γ-crystallins.

**Table 2:** Identified proteins from the WS fraction of 12-week-old Wistar lens with sequence coverage (%) after Mascot search.

**Spot number**	**Identified protein**	**Sequence coverage, %**	**Spot number**	**Identified protein**	**Sequence coverage, %**
1	Grifin	28	**21**	**βB1**	63
2	αA	53	22	βB1	65
3	αA	64	23	βB3	75
4	αA	66	**24**	**βB3**	70
5	αA	53	**25**	**βB2**	50
**6**	**αA**	90	26	βB2	72
7	αA	86	27	βB2	69
8	αA	58	28	αB	42
**9**	**αA_insert_**	39	**29**	**αB**	67
10	βA4	69	**30**	**γS**	74
11	βA4	63	31	γE	63
**12**	**βA4**	63	**32**	**γE**	73
**13**	**βA2**	56	33	γD	67
**14**	**βA3**	62	**34**	**γD**	54
15	βA3	48	**35**	**γF**	76
16	βA3	51	**36**	**γC**	76
17	βA3	51	**37**	**γB**	94
18	βA3	69	**38**	**γA**	61
19	βA3	67	39	FABP	70
20	βA3	62			

Unmodified proteins are indicated by bold fonts.

Two non-crystallin proteins were also identified in the soluble fraction of the lens proteins. One of them is identified as grifin (molecular weight 15,839 Da, accession number O88644 (GRIFN_RAT) at Uniprot. This galectin-related inter-fiber protein is lens-specific, it is located at the interface between lens fiber cells. This protein has been previously described in rat lenses and has a molecular function of sugar binding [41]. In our samples, its level (0.4%–1% of the total protein content) is comparable with the levels of the least abundant crystallins. The other protein found in the rat lens is fatty acid-binding protein (molecular weight 15,059 Da, accession number P55053 (FABP5_RAT) at Uniprot. This protein plays a role of the marker for differentiation of the fiber cells. It was previously found in the mice [25], cow [42], and rat [43] lenses.

### Age-related changes in the crystallin composition

**Figure 2:**
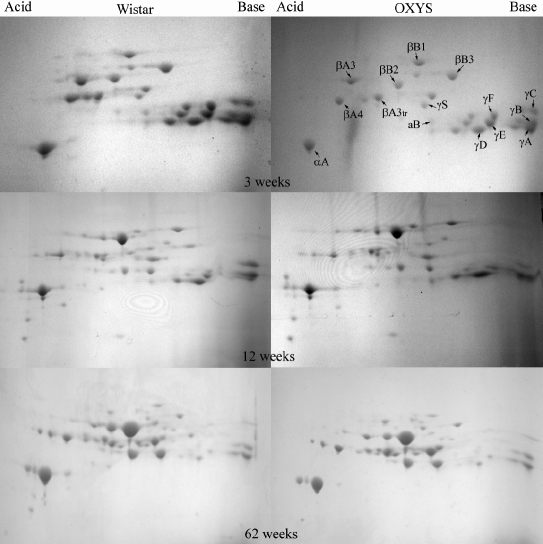
2-DE gels of WS fractions of the lens proteins of 3, 12, and 62-week-old Wistar and OXYS rats. Assignment is given for unmodified crystallins.

**Figure 3:**
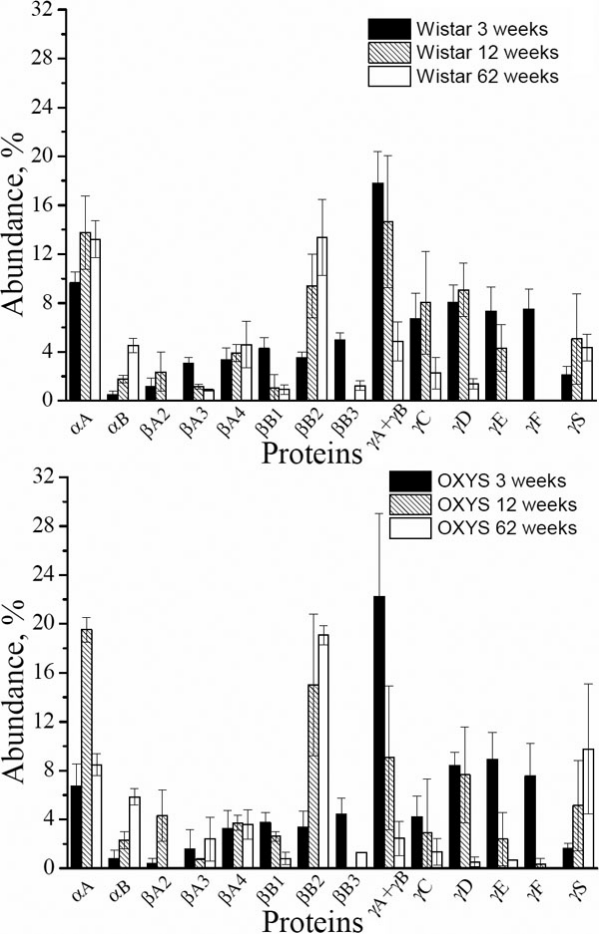
Relative abundances of unmodified soluble crystallins in lenses of Wistar and OXYS rats at the ages of 3, 12 and 62 weeks. Black bars show the values for 3-week-old animals, hatched bars – for 12-week-old animals, and open bars – for 62-week-old animals. Error bars indicate the standard deviation.

**Figure 4:**
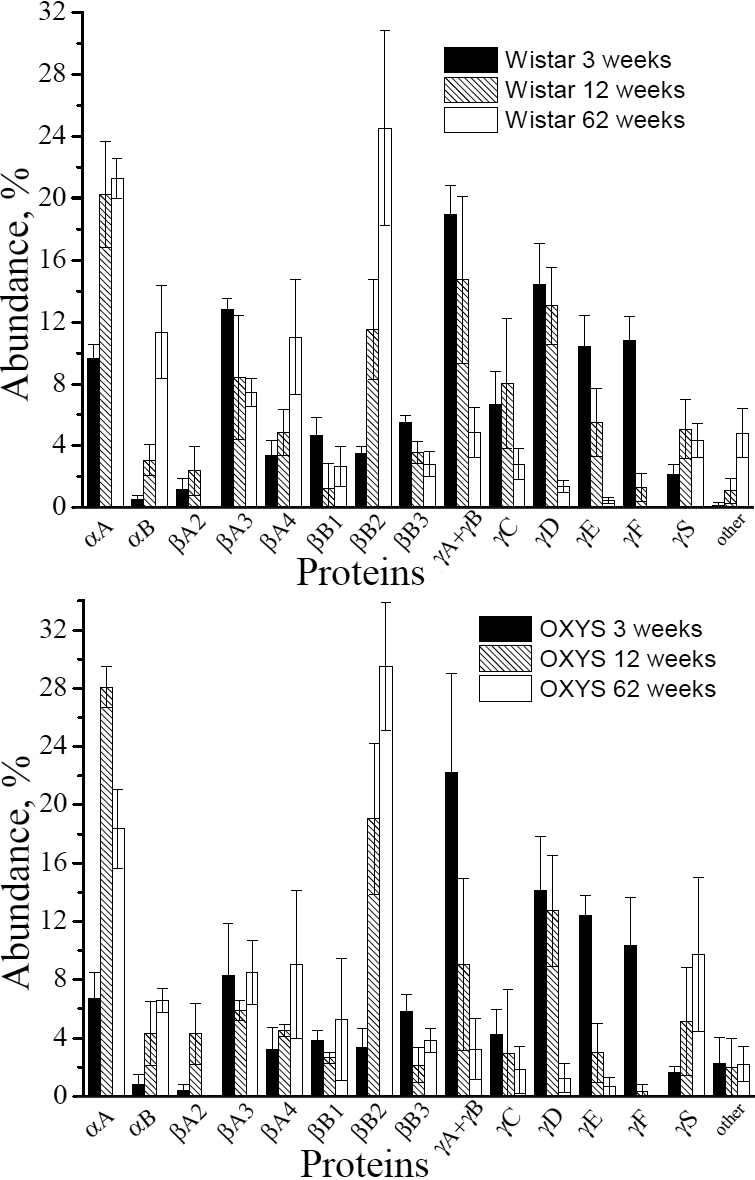
Relative abundances of total soluble proteins in lenses of Wistar and OXYS rats at the ages of 3, 12 and 62 weeks. Black bars show the values for 3-week-old animals, hatched bars – for 12-week-old animals, and open bars – for 62-week-old animals. Error bars indicate the standard deviation.

Age-related changes in the protein composition of rat lenses were monitored using the water-soluble lens proteins from 3-, 12-, and 62-week-old Wistar and OXYS rats. For each age, the lenses from 3-6 animals were taken, the water-soluble protein fraction from each lens was subjected to 2-DE (Figure 2), and the percentage abundance of crystallins observed in gels was determined by the integral density of the corresponding spots. The data obtained for the same age and strain were averaged. Since the spots corresponding to γΑ- and γΒ-crystallin in some gels overlap, the percentage abundances of these crystallins were combined. The average percentage abundance of unmodified proteins are given in Table 3 and Figure 3, and the data on the sum of modified and unmodified crystallins are collected in Table 4 and presented in Figure 4.

**Table 3:** Percentage abundance of unmodified proteins in lenses of Wistar and OXYS rats.

**Age**	**3 weeks**		**12 weeks**		**62 weeks**	
**Strain**	**Wistar**	**OXYS**	**Wistar**	**OXYS**	**Wistar**	**OXYS**
Number of animals	5	6	4	3	4	3
αA	9.6±0.9	6.7±1.8	13.8±3.0	19.5±1.0	13.2±1.5	8.5±0.9
αB	0.5±0.3	0.8±0.6	1.8±0.3	2.3±0.7	4.5±0.6	5.9±0.7
βA2	1.2±0.7	0.4±0.4	3.2±0.4	4.3±2.1	0	0
βA3	3.0±0.5	1.6±1.6	1.2±0.2	0.7±0.1	0.9±0.1	2.4±1.8
βA4	3.3±1.0	3.2±1.3	3.9±0.7	3.7±0.6	4.6±1.9	3.6±1.2
βB1	4.3±0.9	3.7±0.8	1.1±1.1	2.6±0.4	0.9±0.4	0.8±0.5
βB2	3.5±0.5	3.4±1.3	9.4±2.6	15.0±5.8	13.4±3.1	19.1±0.8
βB3	5.0±0.6	4.5±1.3	0	0	1.2±0.4	1.3±0.1
γA + γB	17.8±2.6	24.5±6.6	14.7±5.4	9.1±5.9	4.9±1.6	2.5±1.4
γC	6.7±2.1	4.2±1.7	8.0±4.2	2.9±4.4	2.3±1.3	1.4±1.1
γD	8.0±1.4	8.4±1.1	9.1±2.2	7.7±3.9	1.4±0.4	0.8±0.5
γE	7.4±2.0	8.9±2.2	4.3±1.9	2.4±2.2	0	0.7±0.1
γF	7.5±1.6	7.5±2.7	0	0.9±0.1	0	0
γS	2.1±0.7	1.6±0.4	5.1±1.9	5.1±3.7	4.4±1.1	9.8±5.3

**Table 4:** Percentage abundance of the sum of modified and unmodified proteins in lenses of Wistar and OXYS rats.

**Age**	**3 weeks**		**12 weeks**		**62 weeks**	
**Strain**	**Wistar**	**OXYS**	**Wistar**	**OXYS**	**Wistar**	**OXYS**
Number of animals	5	6	4	3	4	3
αA	9.6±0.9	6.7±1.8	20.2±3.4	28.1±1.4	21.3±1.3	18.4±2.7
αB	0.5±0.3	0.8±0.7	3.1±1.0	4.3±2.2	11.3±3.0	6.6±0.8
βA2	1.2±0.7	0.4±0.4	2.4±1.6	4.3±2.1	0	0
βA3	12.8±0.7	8.3±3.6	8.4±4.0	5.9±0.7	7.5±0.9	8.5±2.2
βA4	3.3±1.0	3.2±1.5	4.9±1.5	4.5±0.4	11.0±3.7	9.0±5.1
βB1	4.7±1.1	3.8±0.7	1.3±1.6	2.6±0.4	2.7±1.3	5.3±4.2
βB2	3.5±0.5	3.4±1.3	11.5±3.2	19.0±5.2	24.5±6.3	29.5±4.4
βB3	5.5±0.4	5.8±1.2	3.6±0.7	2.1±1.2	2.8±0.8	3.8±0.8
γA + γB	19.0±1.8	24.8±3.2	14.7±6.1	9.1±5.9	4.9±1.6	3.2±2.1
γC	6.7±2.1	4.2±1.7	8.0±4.2	2.9±4.4	2.8±1.0	1.8±1.6
γD	14.4±2.6	14.1±3.7	13.0±2.5	12.7±3.8	1.4±0.4	1.2±1.0
γE	10.4±2.0	12.4±1.4	5.5±2.2	3.0±2.0	0.5±0.2	0.7±0.6
γF	10.8±1.5	10.4±3.3	1.3±0.9	0.3±0.5	0	0
γS	2.1±0.7	1.6±0.4	5.1±1.9	5.1±3.7	4.4±1.1	9.8±5.3
others	0.1±0.2	2.3±1.8	1.1±0.8	1.9±2.0	4.8±1.6	2.2±1.2

The most dramatic and rapid age-related changes were registered for γ-crystallins: a fast decay of the concentration is observed for all γ-crystallins (except γS-crystallin). The decay of γ-crystallin in lenses of OXYS rats is faster than that in Wistar. The total content of γA-γF-crystallin in Wistar decays from 61.4% (3 weeks) to 40.6% (12 weeks) to 9.5% (62 weeks), while in OXYS lenses the age dynamics of γA-γF-crystallin is: 65.9% (3 weeks) – 28% (12 weeks) – 7% (62 weeks). The fastest decay is observed for γF-crystallin, which level decreases from 10.8% (3 weeks) to 1.3% (12 weeks) in Wistar and from 10.4% (3 weeks) to 0.3% (12 weeks) in OXYS lenses. At the age of 62 weeks, γF-crystallin have not been detected neither in Wistar nor in OXYS lenses. It is important to note that in all gels the majority of γ-crystallin is present in unmodified state. That likely means that the modifications of γ-crystallins result in their fast insolubilization.

Against a background of the strong γ-crystallin decay, the percentage abundance of other crystallins (with the few exceptions) either increases with age, or remains approximately at the same level. The most pronounced increase is observed for αB- and βB2-crystallin: the percentage of these proteins in 62-week-old lenses is higher than in 3-week-old lenses by approximately an order of magnitude. This effect should be attributed to the enhanced stability of these proteins under oxidative stress. However, the increased expression of αB- and βB2-crystallin in the epithelial cells of the lens also cannot be ruled out. The difference between αB- protein contents in the Wistar and OXYS lenses at 3 weeks and 12 weeks is insignificant, but at 62 weeks the percentage abundance of αB-crystallin in Wistar is twice as large as in OXYS lenses, mostly due to the high abundance of modified αB-crystallin in the WS fraction of proteins from the Wistar lenses. The most plausible explanation of this observation is that at the age of 62 weeks, the modifications of αB-crystallin in OXYS lenses become too severe, and they undergo insolubilization.

An interesting dynamics is observed for αA-crystallin. During the period from 3 weeks to 12 weeks, the sum of modified and unmodified αA-crystallin in lenses of Wistar rats increases from 9.6% to 20.2%, and in OXYS from 6.7% to 28.1%. At 62 weeks, the percentage of αA-crystallin in Wistar remains approximately the same as in 12 weeks, while in OXYS lenses it decreases to 18.4%. At this age, unmodified αA-crystallin constitutes about 60% of the total amount of αA-crystallin in Wistar lenses, and only approximately 45% in OXYS.

## Discussion

2-DE maps of young rat lenses obtained in the present work are in a good agreement with the previously reported proteome maps of lenses of Sprague-Dawley rats [29] and FVB/N mice [25]. The position, masses and pIs of crystallins from rats of different strains and from mice are very similar, which indicates the absence of differences in the initial steps of rodent’s lens development. The general content of WS proteins in young lenses from Wistar and Sprague-Dawley rats is also similar: approximately 10% of α-crystallin, 30% of β-crystallin, and 60% of γ-crystallin [29]. Nevertheless, the percentage abundances of some individual proteins are different. The most significant difference was found for αB-crystallin, which content in 3-week-old Wistar and OXYS lenses (0.5%–0.8%) is much lower than that in 16-day-old Sprague-Dawley lens (approximately 4% [29]).

The time evolution of the lens protein composition includes the changes in the contribution of α-, β-, and γ-crystallin families into total protein content as well as in the abundance of individual crystallins. The major factor, which influences the protein composition in WS fraction, is the insolubilization of γ-crystallin. This process occurs in both Wistar and OXYS lenses (although in OXYS it proceeds faster) and results in the decrease of the γA-γF-crystallin percentage abundance down to 7%–10% to the age of 62 weeks. γ-Crystallins are fully presented in rodent lenses, and their intensive degradation during aging has been reported for both mice [25] and rats [44]. In human lenses, there are three types of γ-crystallin: γC-, γD-, and γS-crystallin [45]. It has been shown [17] that the concentration of γC- and γD-crystallin in human lens also decreases with age. The major process causing the γ-crystallin insolubilization might be the extensive deamidation of crystallins, which enhances the acidity of proteins [18], and the truncation [46].

Very likely that the insolubilization of γ-crystallin is the main cause of the age-related changes in WIS/WS ratio (Table 1): at the age of 3 weeks, WS proteins constitute 30%–40% of the total lens weight, and WIS proteins – only 3%–5%; at the age of 62 weeks, the contribution of WS fraction decreases to approximately 25%, and that of WIS fraction increases to about 20%. The age-related changes in WIS/WS ratio in the Wistar lens has been recently reported [5]: from the age of 16 days to 1 year, this ratio increases by approximately a factor of four, which is in a good agreement with our data. We should note that the absolute values of WIS/WS ratio in work [5] were significantly lower than in the present work – probably, due to the different procedures of the WS and WIS fraction measurement.

The insolubilization of the lens protein is often referred to as one of the main factors of the cataract development: insoluble proteins may form large aggregates causing light scattering and lens opacification. Our results suggest that the increase of the WIS fraction is the age-specific rather than cataract-specific process: the WIS/WS ratio in Wistar and senescence-accelerated OXYS rat lenses is approximately the same for all ages, though the onset of the cataract in OXYS rats occurs much earlier [39].

From 3 weeks to 12 weeks, the most significant increase in the percentage abundance was found for both α-crystallin and βB2-crystallin. In the lens, αA-crystallin plays a very important role due to its chaperone activity [8,18,47]: with aging, α-crystallin binds partially unfolded β- and γ-crystallins, thereby preventing their further aggregation and precipitation, and delaying lens opacification [9,47]. Thus, the increase of the α-crystallin content can be attributed to the compensatory reaction of the lens to the oxidative stress, which causes the enhanced expression of α-crystallin. To confirm this assumption, we performed the comparative measurement of the α-crystallin expression in young (3-week-old) Wistar and OXYS lenses using real time PCR. The expression of α-crystallin was measured for lenses of 7 Wistar and 7 OXYS rats using the housekeeping gene *Rpl30* as a reference gene (see Experimental section), the results were averaged. It has been found that the expression of both αA- and αB-crystallin (E_αA_ and E_αB_) in OXYS lenses is twice as large as that in Wistar lenses: E_αA_(Wistar)=2.45±0.28; E_αA_(OXYS)=4.87±0.27; E_αB_(Wistar)=3.04±0.21; E_αB_(OXYS)=6.11±0.53. Since tissues in OXYS rats are more exposed to the oxidative stress than ones in Wistar rats, the compensatory reactions should be stronger. Thus, the more significant growth of the α-crystallin content in OXYS lenses in comparison with that in Wistar rats should be attributed to the enhanced expression of α-crystallin as a compensatory reaction to the oxidative stress. PTMs cause breaches in the protein folding, and accumulation of proteins with the “wrong” folding can initiate unfolded protein response, which implies an accelerated degradation of “wrong” proteins as well as a deceleration of protein synthesis [48]. This mechanism can explain age-related decrease in synthesis of some proteins.

The content of αA-crystallin in Wistar lenses at 62 weeks is approximately the same as at 12 weeks, whereas in OXYS lenses it decreases with age. This decrease is especially pronounced for unmodified αA-crystallin, which percentage abundance in 62-week-old OXYS lenses is less than a half from that in Wistar lenses of the same age. This finding correlates with the recent report on very low αA-crystallin expression in old OXYS lenses [39], which likely can be attributed to the damages affecting the epithelial cell functioning. Another possible explanation of the faster αA-crystallin loss during aging than that of αB-crystallin is that αA-crystallin is more vulnerable to the oxidative stress. This hypothesis is confirmed by the recent studies of α-crystallin distribution in the bovine lenses. It has been shown [7] that αB-crystallin is stable on both the periphery and the nucleus of the bovine lens, which means that this protein should accumulate with age. On the contrary, intact αA-crystallin was found only on the periphery of bovine lens, where new proteins are synthesized [18,47]. The three major truncated forms αA_1–101_, αA_1–65_, αA_1–58_ were observed across the whole section and especially in the nucleus where older proteins accumulate with age [7].

The content of βB2-crystallin in WS fraction of the lens proteins undergoes a stable growth throughout the lifespan of both Wistar and OXYS rats, demonstrating the high stability of this protein under oxidative stress. It was suggested that with the loss of αA-crystallin, it might be substituted in the lens structure by βB2-crystallin [45].

In summary, the results of this work show that the most pronounced age-related changes in the protein composition of the rat lens are the increase of WIS/WS ratio with aging, the fast insolubilization of γA-γF-crystallins, and the increase of αB- and βB2-crystallin abundance in the WS protein fraction during the lens growth. The percentage abundance of αA-crystallin in WS fraction grows only in relatively young lenses. The major differences between Wistar and OXYS lenses are the faster decay of the content of γA-γF-crystallin in OXYS lenses, and the significant decrease of unmodified αA-crystallin abundance in old OXYS lenses. These differences can be attributed to the cataract-specific changes in the protein composition of the lens. More detailed information on the cataract-specific processes in the lens can be obtained from the analysis of the content of the WIS fraction of lens proteins, and from the comparative study of post-translational modifications of proteins in Wistar and OXYS lenses. This work is currently in progress in our laboratory.
